# The spectrum of polypoidal choroidal vasculopathy in Caucasians: clinical characteristics and proposal of a classification

**DOI:** 10.1007/s00417-020-04844-z

**Published:** 2020-08-18

**Authors:** Elon H. C. van Dijk, Danial Mohabati, Simona Veselinovic, Wing H. Chung, Greet Dijkman, Camiel J. F. Boon

**Affiliations:** 1 Department of Ophthalmology, Leiden University Medical Center, Albinusdreef 2, 2333 ZA Leiden, The Netherlands; 2 Department of Ophthalmology, Amsterdam University Medical Centers, University of Amsterdam, Amsterdam, The Netherlands; 3 Department of Ophthalmology, Leiden University Medical Center, P.O. Box 9600, 2300 RC Leiden, The Netherlands

**Keywords:** Aneurysmal type 1 neovascularization, Caucasians, Classification, Clinical characteristics, Polypoidal choroidal vasculopathy, Sub-retinal pigment epithelium neovascularization

## Abstract

**Purpose:**

To describe the clinical characteristics and outcome of polypoidal choroidal vasculopathy (PCV), also known as aneurysmal type 1 (sub-retinal pigment epithelium (RPE)) neovascularization, in Caucasian patients.

**Methods:**

Single-centre study in 66 Caucasian patients with a diagnosis of PCV based on optical coherence tomography scan and indocyanine green angiography. Clinical characteristics and multimodal imaging were collected and assessed by an experienced retina specialist.

**Results:**

This study involved 74 eyes of 66 patients with PCV, with a mean age at onset of 73 years and a female preponderance of 66%. The mean number of polypoidal lesions per eye was 1 (range: 1–5 lesions), out of which 75% was located in the macula and 19% in the peripapillary region. Of the 74 eyes, 37 eyes (50%) had PCV associated with a drusenoidal neovascular age-related macular degeneration (AMD) phenotype (PCV-AMD) and 18 eyes (24%) had PCV associated with non-polypoidal type 1 choroidal neovascularization/branching vascular network (PCV-BVN) without signs of drusenoidal AMD, while 19 eyes (26%) had idiopathic, isolated PCV (iPCV). The mean subfoveal choroidal thickness measured in 22 patients was 245 μm (range: 71–420 μm). In 51% of patients, the initially performed therapy showed good anatomical recovery (resolution of intra- and subretinal fluid).

**Conclusions:**

A spectrum of PCV (aneurysmal type 1/sub-RPE neovascularization) can be seen in Caucasian patients. PCV associated with a drusenoidal neovascular AMD phenotype in Caucasians is phenotypically and presumably pathophysiologically more associated with neovascular AMD (PCV-AMD: type A PCV). However, this may not be the case for patients with PCV with non-polypoidal type 1 choroidal neovascularization or BVN and no signs of drusenoidal AMD (PCV-BVN: type B PCV), and for patients with idiopathic PCV without associated drusen or BVN (iPCV; type C PCV). Most patients have a thin choroid, even when drusen are absent. For the entire patient group, a moderate anatomical recovery was observed after treatment.

**Electronic supplementary material:**

The online version of this article (10.1007/s00417-020-04844-z) contains supplementary material, which is available to authorized users.

## Introduction

Idiopathic polypoidal choroidal vasculopathy (PCV) was first described in 1990 by Yannuzzi et al. as peculiar polypoidal subretinal lesions that were associated with haemorrhagic detachments of the retinal pigment epithelium (RPE) [1]. Later, Yannuzzi suggested that PCV may be a self-contained clinical entity, which involves elevated reddish to orange lesions on fundus examination, dilated inner choroidal vessels, and polypoidal vascular structures beneath a RPE detachment [2].

Recently, controversy has arisen regarding the definition of PCV and its association with neovascular age-related macular degeneration (nAMD) [2–5]. PCV and nAMD have been found to have similar environmental risk factors and to share molecular and genetic determinants involving the complement pathway [6]. Furthermore, several studies have shown that polypoidal lesions in PCV are often associated with a branching vascular network (BVN) of subretinal neovascularization between the RPE and Bruch’s membrane, indicating that PCV could be a variant of type 1 neovascularization of any origin [4, 7]. Patients with type 1 (sub-RPE) subretinal neovascularization can have an aneurysmal dilation (the ‘polyp’), often at the edge of the neovascularization. This aneurysmal dilation is also known with the term PCV, which strictly speaking is a misnomer because we have learned that neovascular lesions that characterize PCV do not arise directly from the choroid [3]. The aneurysmal dilation or PCV typically shows as a well-circumscribed hyperfluorescent lesion already in the early phase on indocyanine green angiography (ICGA). The non-aneurysmal part of the type 1 neovascular lesion is often referred to as a BVN, which can therefore be present in every macular neovascular condition [3, 4, 6]. In comparison with nAMD without PCV, patients with PCV may show relative resistance to anti-vascular endothelial growth factor (VEGF) treatment [8, 9]. Based on imaging studies, some authors have suggested that the term PCV should be reserved for patients in whom findings characteristic for AMD, such as drusen, pigmentary changes, and geographic atrophy, are absent [7, 10, 11].

PCV has been studied mostly in Asian populations. These studies have shown a relatively high prevalence of PCV of up to 61.6% in Asian patients who presumably had nAMD [10, 12, 13]. However, this prevalence has been shown to be only 8.7% in Caucasian patients with a suspicion of nAMD [14, 15], although a recent study described a prevalence of up to 31.9% [16]. A study in which the occurrence of PCV was compared directly between an Asian and non-Asian nAMD cohort showed a large difference: 48% of Japanese patients and 9% of French nAMD patients had PCV [17]. Despite the fact that drusen have traditionally been considered hallmark and even mandatory lesions for the diagnosis of AMD [18], we have also described a spectrum of nAMD in elderly Caucasian patients without drusen in the fellow eye, in whom 5 patients (10%) were shown to have evidence of PCV in the affected eye [19]. This may indicate that the aetiology of non-drusen-associated nAMD and PCV may be different from drusenoidal neovascular subtypes [19]. Little is known about the risk factors, clinical characteristics, and treatment outcome of PCV in Caucasians, as previously conducted studies had small sample sizes [20–23].

The purpose of this study is to describe the anatomical characteristics and the clinical course of PCV in the largest Caucasian population published so far. We show that there are different clinical manifestations of PCV in Caucasians, and propose a PCV classification into 3 subtypes, comparing our findings in the present study population to the available data on Caucasian nAMD patients, and to previously published characteristics of Asian PCV patients.

## Methods

### Study population

A retrospective review of medical charts was performed for patients who visited the Department of Ophthalmology of Leiden University Medical Center (Leiden, the Netherlands), a tertiary referral centre for medical retina diseases in The Netherlands. This study adhered to the tenets of the Declaration of Helsinki and all federal laws in The Netherlands, and approval for this retrospective study was obtained from the Medical Ethical Committee of Leiden University Medical Center. From 2000 to 2019, characteristics and multimodal imaging from consecutive patients who were suspected to have PCV were collected, based on the following: (1) a pink-orange subretinal nodular lesion on fundoscopy; (2) a peaked/dome-shaped elevation of the RPE, often with a hyperreflective subretinal accumulation beneath it; (3) non-response or only partial response to anti-VEGF treatment in patients with macular neovascularization without evidence of another diagnosis than AMD. All images were obtained from routine eye examinations and included colour fundus photographs (Topcon Corp., Tokyo, Japan), optical coherence tomography (OCT) scans with either the time-domain (Cirrus OCT Carl Zeiss Meditec, Dublin, CA, USA) or spectral-domain OCT (SD-OCT) (Spectralis HRA+OCT, Heidelberg Engineering GmbH, Heidelberg, Germany) device, ICGA (Spectralis HRA+OCT), and fluorescein angiography (FA) (performed with either the Topcon fundus camera or the Spectralis HRA+OCT camera).

**Fig. 1:**
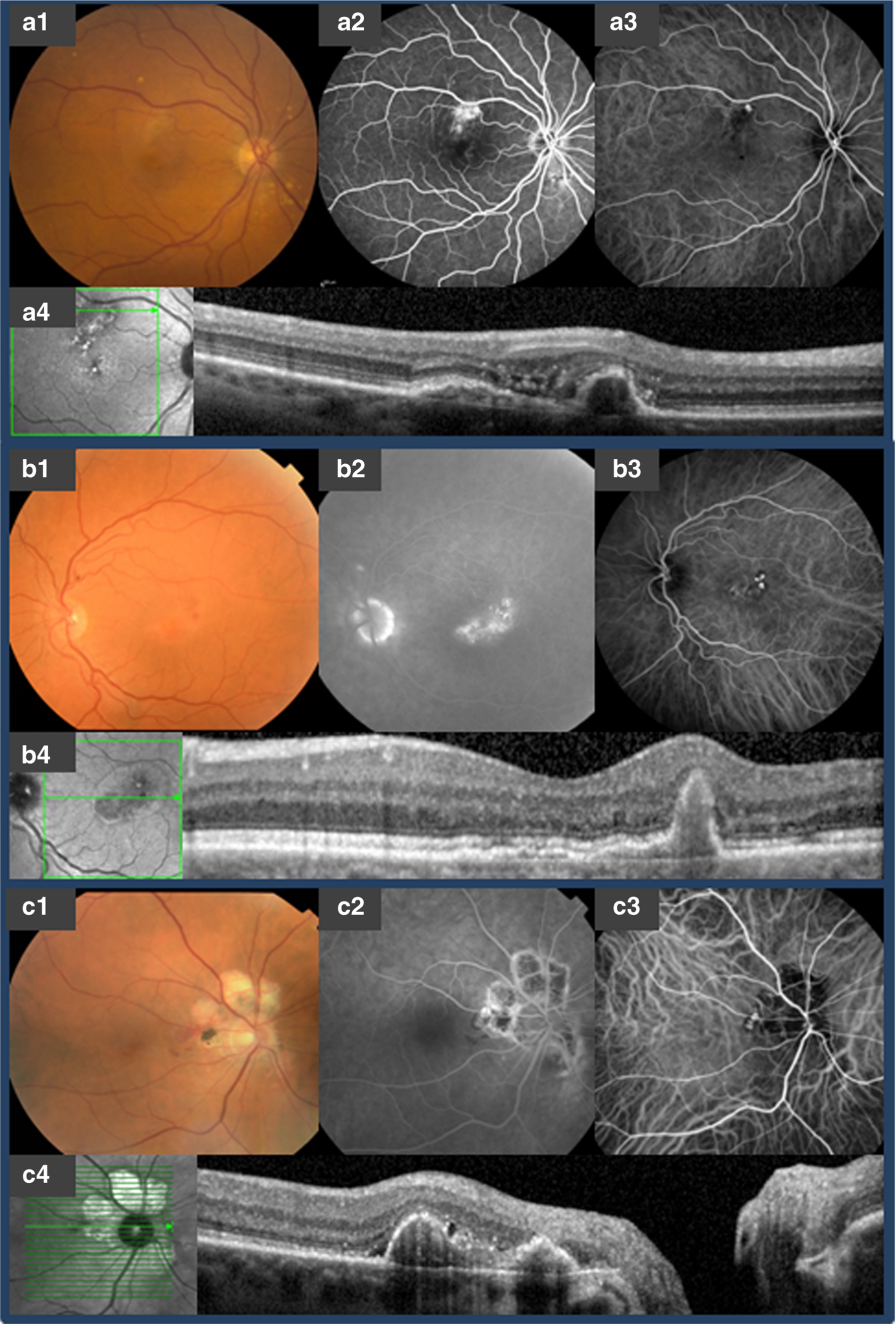
Multi-modal imaging of subtypes of polypoidal choroidal vasculopathy. A, multimodal imaging in a patient with polypoidal choroidal vasculopathy (PCV) associated with drusenoidal neovascular age-related macular degeneration (AMD). A1, colour fundus photography shows drusen, central hyperpigmentation, and an orange nodule superiorly in the macula. A2, late-phase fundus fluorescein angiography (FA) shows leakage of fluorescein in an occult neovascular pattern. A3, indocyanine green angiography (ICGA) shows a single solitary polypoidal lesion without a vascular network. A4, spectral-domain optical coherence tomography (SD-OCT) shows subretinal fluid and a peaked elevation of the retinal pigment epithelium (RPE) that represents the polypoidal lesion. B, multimodal imaging in a patient with non-AMD associated PCV in combination with a branching vascular network (BVN). B1, colour fundus photography shows an orange nodule within the macula, without signs of AMD. B2, late-phase FA shows leakage of fluorescein that corresponds with the polypoidal lesion. B3, ICGA shows 3 solitary polypoidal lesions associated with a vascular network nasally. B4, SD-OCT showing a dome-shaped RPE detachment with a hyperreflective subretinal accumulation that corresponds with the polypoidal lesion. This polypoidal lesion is associated with a double-layer sign on the nasal side, which represents a BVN. C, multimodal imaging in a patient with idiopathic PCV. C1, colour fundus photography showing peripapillary atrophy associated with a brown-reddish nodule within the papillomacular bundle. C2, Late-phase FA showing minimal leakage of fluorescein that corresponds with the polypoidal lesion and window defects that correspond with peripapillary atrophy. C3, ICGA shows a solitary polypoidal lesion without a vascular network. C4, On SD-OCT 2 dome-shaped RPE detachments with a hyperreflective subretinal accumulation that correspond with the polypoidal lesions, can be observed.

Only patients of Caucasian ethnicity were included in the study if they met all of the following inclusion criteria: (1) a diagnosis of PCV was established when single or multiple focal nodular areas of hyperfluorescence were visible within the first 6 min after dye injection on ICGA, with or without a choroidal interconnecting vascular network, together with characteristic nodular hyperfluorescent structures on FA [3, 7, 15, 24–26]; (2) availability of clinical information and imaging for at least 1 visit after initial diagnosis and treatment (Fig. 1). Exclusion criteria were any of the following: (1) insufficient clinical data at diagnosis; (2) insufficient quality of multimodal imaging; (3) evidence of diagnoses other than either AMD or idiopathic PCV, with a possible pathophysiological background for PCV, such as central serous chorioretinopathy, as we aimed to include only patients who had PCV as ‘primary disease’.

### Clinical examinations and classification

Patients who met the inclusion criteria were graded in 3 subtypes of PCV based on the presence of drusen, CNV, and BVN on multimodal imaging which was assessed by an experienced retina specialist (CJFB). Since no clear definition for BVN was found in literature, we defined this as a network on ICGA within the first 6 min after injection of indocyanine green that corresponded to a ‘double-layer sign’ on OCT, corresponding to a shallow RPE detachment separated from Bruch’s membrane by an area of moderate reflectivity [27, 28], without the presence of extensive (late) leakage on FA [7]. This is in contrast to the peaked/dome-shaped/‘thumb-like’ polyp [6]. On ICGA, a non-polypoidal type 1 neovascular lesion does not show early well-circumscribed hyperfluorescence, which is in contrast to a polypoidal lesion [6].

The cases were subsequently divided in 3 groups, based on the previous description of Coscas et al. [7], taking into account that a CNV or a BVN could also occur in patients in whom no signs of AMD are present. These 3 groups included the following: (1) PCV within the context of nAMD with the presence of ≥ 5 drusen with a diameter of ≥ 63 μm in the affected or fellow eye; (2) PCV in combination with either a non-polypoidal type 1 CNV or BVN without signs of AMD (i.e. the presence of < 5 either hard or soft drusen with a diameter of ≥ 63 μm) in the affected or fellow eye; (3) idiopathic PCV without drusen in the affected or fellow eye, and without signs of either CNV or BVN in the affected eye.

**Fig. 2:**
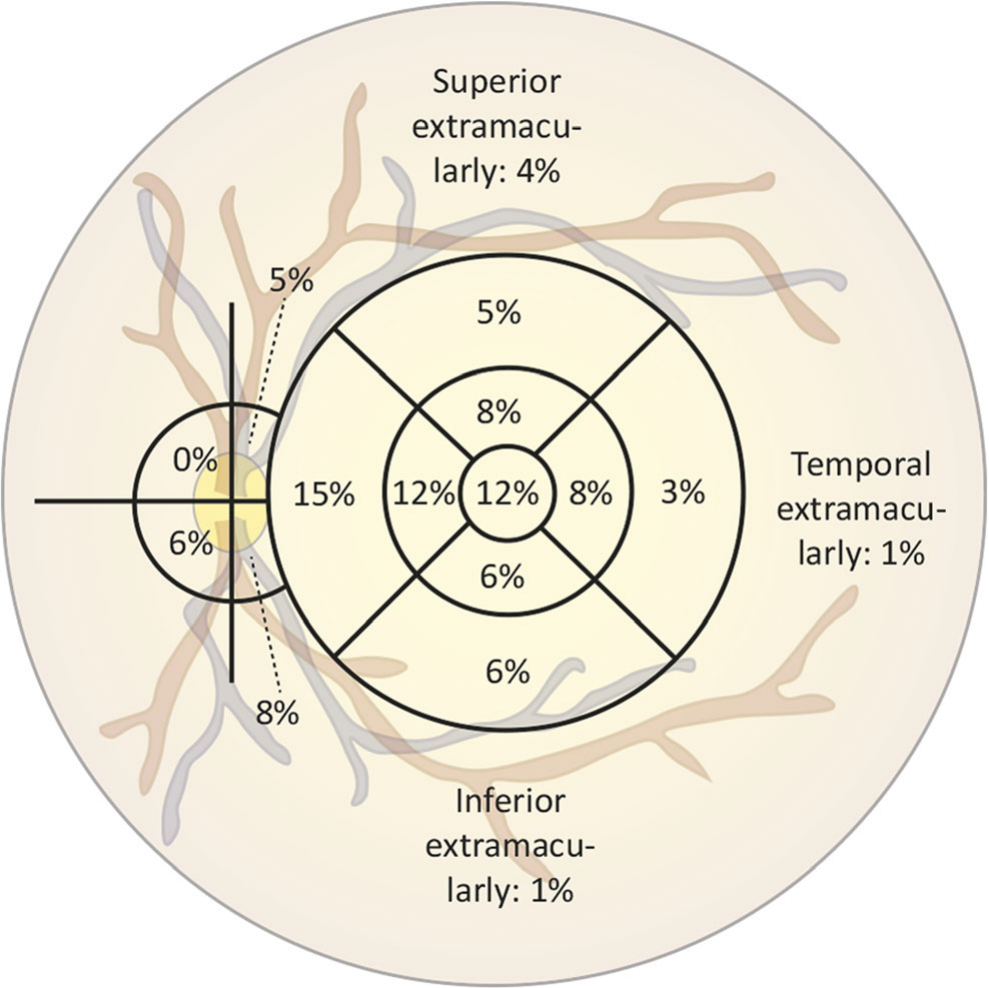
Distribution of 144 polypoidal lesions among 74 eyes of Caucasian polypoidal choroidal vasculopathy patients

The number, shape, and location of the polypoidal lesions were subsequently assessed. The following shapes were distinguished: isolated polypoidal lesions, string-like polypoidal lesions (at least 3 solitary polyps in line) [29], and a cluster of grape-like polypoidal lesions [30]. The presence and location of polypoidal lesions were determined on ICGA imaging within 6 min after injection of indocyanine green [25]. After determination of the exact location of the fovea using SD-OCT and fundus photography, ICGA images were placed on top of the fundus photograph and an Early Treatment of Diabetic Retinopathy Study (ETDRS) grid with 3 solid circles of sizes 7200, 3600, and 1200 μm was added. In those cases in which SD-OCT was not available, the location of the fovea was estimated using the time-domain OCT. The presence of polypoidal lesions for each anatomical region is depicted in Fig. 2.

For both eyes, information on the presence of macular haemorrhages and retinal atrophy were obtained at baseline. The origin of polypoidal lesions was studied by analysing possible interruptions of Bruch’s membrane in those patients for whom SD-OCT of sufficient quality was available. Pachyvessels (pathologically dilated Haller layer with attenuation and thinning of the choriocapillaris and Sattle layer), and subfoveal choroidal thickness (distance from the outer part of the hyperreflective RPE layer to the hyperreflective line of the inner surface of the sclera), were studied in patients for whom OCT imaging of sufficient quality was available [3]. A subfoveal choroidal thickness of > 350 μm was considered to be a pachychoroid in our relatively old patient group [31].

### Data collection at baseline and follow-up

The following information was obtained for the visit at which PCV was diagnosed (baseline): age at onset, best-corrected visual acuity (BCVA; in Snellen letters, and subsequently converted to ETDRS letters), subjective symptoms before diagnosis of PCV, initial diagnosis at the referring ophthalmologist before suspicion of PCV at our centre, treatments before suspicion of PCV and after diagnosis of PCV, and total duration of follow-up. At the first follow-up visit and visits at on average at 6, 12, and 24 months after first treatment, the following information was obtained: BCVA, evaluation of subjective symptoms, performed treatments, treatment effect (defined as complete resolution of either intraretinal fluid or subretinal fluid) on OCT), and disease recurrence. Patients were noted as lost to follow-up, when no disease reoccurrence was observed at clinical evaluation in our centre after treatment and patient was thereafter sent back to the referring ophthalmologist.

### Statistical analysis

Statistical analysis was performed using IBM SPSS Statistics, version 23 (IBM, Armonk, NY, USA). A one-way ANOVA test was performed for normal distributed numeric data, and both a Wilcoxon signed rank test and a Kruskal-Wallis test were used for not normally distributed numeric data. For the analysis of the categorical data, a chi-square test or a McNemar’s test, or a Fisher’s exact test was used. A *P* value of < 0.05 was considered statistically significant for all performed analyses.

## Results

### Clinical characteristics

A total of 74 eyes of 66 consecutive patients, with a mean age of 73 years (range: 51–90), with PCV were included in this study. In 44 of these eyes a pink-orange subretinal nodular lesion on fundoscopy, and/or a peaked/dome-shaped elevation of the RPE, usually with a hyperreflective subretinal accumulation beneath it, was found at baseline, whereas in 30 eyes a macular neovascularization without evidence of another diagnosis than AMD that either did not respond or only partially responded to previous anti-VEGF treatment was seen. The characteristics of the participants are summarized in Table 1. Table 1 also shows the BCVA of 3 different groups of PCV patients, for whom a follow-up of 24 months was available. At baseline, the median BCVA was 66.3 ETDRS letters and showed no differences between the 3 groups that comprised of a relatively low number of patients (*P* = 0.082).

**Table 1:** Baseline characteristics of all Caucasian polypoidal choroidal vasculopathy patients

	Total population (eyes)	Subtypes of PCV			*P* value
		PCV in context nAMD (drusen; type A)	PCV and non-polypoidal type 1 CNV/BVN (type B)	Idiopathic PCV (type C)	
Patients (eyes)	66 (74)	33 (37)	17 (18)	16 (19)	
Mean age at onset in years (range)	73 (51–90)	73 (51–90)	70 (57–90)	76 (58–89)	0.165^a^
BCVA in ETDRS letters (±SD)	66.3 (2.3)	63.8 (3.5)	62.1 (4.3)	75.1 (4.1)	0.082^a^
Male gender (%)	25 (34)	11 (30)	9 (50)	5 (26)	0.239^b^
Subjective symptoms (%)					0.292^c^
Blurred vision	36 (48.6)	22 (59.5)	8 (44.4)	6 (31.6)	
Spot in the field of vision	10 (13.5)	2 (5.4)	4 (22.2)	4 (21.1)	
Metamorphopsia	9 (12.2)	4 (10.8)	3 (16.7)	2 (10.5)	
Other	18 (24.3)	8 (21.6)	3 (16.7)	7 (36.8)	
None	1 (1.4)	1 (2.7)	-	-	
Median follow-up duration in days (range)	343 (14–1704)	344 (14–816)	281 (35–1704)	349 (38–840)	0.919 ^d^

^a^One-way ANOVA

^b^*X*^2^ test

^c^Fisher’s exact test

^d^Kruskal-Wallis test

*BCVA*, best-corrected visual acuity; *ETDRS*, Early Treatment of Diabetic Retinopathy Study; *n*, number of eyes; *PCV*, polypoidal choroidal vasculopathy; *SD*, standard deviation

### Characteristics of polypoidal lesions

In the 74 eyes, a total number of 144 polypoidal lesions could be identified (Table 2), which all could be found below the RPE, compatible with a type 1 neovascularization. Multiple lesions were seen in 30 eyes (41%; range: 1 to 5 lesions; median: 1 lesion). Eighty-six of all polypoidal lesions (60%) were solitary, 46 (32%) were in cluster(s), and 10 (7%) occurred in a string-like configuration (Fig. 2). Thirty-seven eyes (50%) could be classified as having PCV within the context of nAMD, 18 eyes (24%) as having PCV in combination with a non-polypoidal type 1 CNV or BVN but without signs of AMD in both eyes, and 19 eyes (26%) as having idiopathic polyps without either drusen in both eyes or signs of either CNV or BVN in the fellow eye (Fig. 1). The exact location of the polypoidal lesion was determined by using an SD-OCT-guided grid, with which 75% of the 144 polypoidal lesions were located within the macular region. The distribution of polypoidal lesions is depicted in Fig. 2.

**Table 2:** Clinical characteristics of all Caucasian polypoidal choroidal vasculopathy patients

	Total population (eyes)	PCV in context nAMD (drusen; type A); *n* = 37	PCV and non-polypoidal type 1 CNV/BVN (type B); *n* = 18	Idiopathic PCV (type C); *n* = 19	*P* value
Number of polypoidal lesions, median (range)	1 (1–5)	1 (1–5)	1 (1–5)	1 (1–3)	0.238^a^
Macular haemorrhage					1.000 ^b^
Yes	13 (17.6)	7 (18.9)	3 (16.7)	3 (15.8)	
Bilateral PCV, *n* (%)					0.637 ^b^
Yes	16 (21.6)	8 (21.6)	2 (11.1)	6 (31.6)	
No	51 (68.9)	26 (70.3)	14 (77.8)	11 (57.9)	
Unknown	7 (9.5)	3 (8.1)	2 (11.1)	2 (10.5)	

^a^Type 2 and type 3 neovascularization did not occur in this cohort

^b^Fisher’s exact test

*BVN*, branching vascular network; *CNV*, choroidal neovascularization; *n*, number of eyes; *nAMD*, neovascular age-related macular degeneration; *PCV*, polypoidal choroidal vasculopathy

Since the majority of our patients have been included in the pre-(SD-)OCT era, an SD-OCT was available for only 23 eyes (31%). However, in 6 cases it was not possible to analyse the interruptions of Bruch’s membrane due to either masking shadowing effects caused by either overlying sub-RPE abnormalities (4 eyes) or an incomplete depiction of the polypoidal lesion on OCT (2 eyes). Three eyes (18%) had an interruption of more than 30 μm, with apparent direct communication between the polypoidal lesion and the underlying choroidal vasculature. For 22 affected eyes, the subfoveal choroidal thickness could be studied on SD-OCT, showing a mean of 245 μm (range: 71–420 μm). Despite the fact that most of these patients have a relatively thin choroid, (relative) pachyvessels were seen in 6 (27.3%) of these eyes. Of the 21 patients with a subfoveal choroidal thickness < 350 μm, the presence or absence of drusen in the affected and fellow eye could be determined in 17 patients (81.0%). Fifteen of these patients (88.2%) did not have any drusen. One patient (5.9%) had extensive reticular pseudodrusen in both eyes, and the second patient had large, confluent sub-RPE drusen in the fellow eye.

### Treatment outcome

Thirty-five eyes out of 73 patients with available information (47%) were treatment-naive at the moment of referral to our tertiary referral centre. Out of the 38 eyes (51%) which received treatment before referral, 30 patients (79%) had received intravitreal anti-VEGF injections; 4 patients had received a combination therapy of PDT and thermal laser (11%); 2 patients (5%) had been treated with a combination therapy of both PDT and intravitreal anti-VEGF injections, and thermal laser; and 2 patients (5%) had been treated with a combination therapy of PDT and intravitreal anti-VEGF injections.

Performed treatments for PCV during follow-up are shown in Table 3. After the diagnosis of PCV was established, in this historical cohort thermal laser was performed in 25 patients (34%), a combination of PDT and intravitreal injection with anti-VEGF in 25 patients (34%), and 24 patients (31%) received either other treatment combinations as initial therapy or patients received no treatment. Additional treatment was required in 29 patients (39%) at first visit after initial therapy, in 17 patients (33%) at 6 months after initial therapy, and in 10 patients (26%) at 12 months after initial therapy, respectively. Intravitreal injection with anti-VEGF medication was the most frequently performed additional therapy (Supplementary Table 1).

**Table 3:** Performed treatments at different follow-up visits for all Caucasian polypoidal choroidal vasculopathy patients

	Initial treatment (*n* = 74)	2 months (*n* = 74)	6 months (*n* = 51)	12 months (*n* = 39)	Total treatment
Anti-VEGF, *n* (%)	2 (2.7)	15 (20.3)	9 (17.6)	5 (12.8)	2 (2.7)
Bevacizumab, *n* (# injections per eye)	1 (2)	10 (3)	3 (3)	3 (5)	2 (2)
Ranibizumab, *n* (# injections per eye)	-	3 (2)	5 (3)	1 (NA)	-
Aflibercept, *n* (# injections per eye)	1 (2)	1 (2)	1 (6)	1 (3)	1 (2)
PDT, *n* (%)	13 (17.6)	4 (5.4)	2 (3.9)	-	5 (6.8)
Normal settings PDT, n	8	1	1		2
Reduced settings PDT, n	3	2	1		1
Unknown settings PDT, n	2	1	-		2
Thermal laser, *n* (%)	25 (33.8)	5 (6.8)	1 (2)	2 (5.1)	21 (28.4)
Anti-VEGF and PDT	25 (33.8)	3 (4.1)	2 (4.1)	2 (5.1)	28 (37.8)
Bevacizumab, *n* (# injections per eye)	20 (2)	1 (2)	2 (2)	2 (2)	32 (2)
Ranibizumab, *n* (# injections per eye)	3 (3)	1 (NA)	-	-	11 (3)
Aflibercept, *n* (# injections per eye)	2 (3)	1 (NA)	-	-	5 (4)
Normal settings PDT, *n*	21	2	-	1	25
Reduced settings PDT, *n*	4	1	1	-	9
Unknown settings PDT, *n*	-	-	1	1	-
Anti-VEGF and thermal laser, *n* (%)	3 (4.1)	3 (4.1)	2 (3.9)	1 (2.6)	5 (6.8)
Bevacizumab, *n* (# injections per eye)	2 (2)	3 (4.3)	2 (4)	1 (3)	9 (2)
Ranibizumab, *n* (# injections per eye)	1 (1)	-	-		1 (1)
Aflibercept, *n* (# injections per eye)	-	-	-		-
PDT and thermal laser, *n* (%)	2 (2.7)	-	-	-	5 (6.8)
Normal settings PDT, *n*	1				5
Reduced settings PDT, *n*	-				1
Unknown settings PDT, *n*	1				1
Anti-VEGF and PDT and thermal laser, *n* (%)	-	-	1 (2)	-	6 (8.1)
Bevacizumab, *n* (# injections per eye)			1 (3)		8 (3)
Ranibizumab, *n* (# injections per eye)			-		2 (2)
Aflibercept, *n* (# injections per eye)			-		1 (2)
Normal settings PDT, *n*			1		6
Reduced settings PDT, *n*			-		1
Unknown settings PDT, *n*			-		2
None, *n* (%)	4 (5.4)	45 (60.8)	34 (66.7)	29 (74.4)	2 (2.7)

*BCVA*, best-corrected visual acuity; *FU*, follow-up; *n*, number of eyes; *NA*, unknown/not available; *PDT*, photodynamic therapy; *VEGF*, vascular endothelial growth factor; #, number

## Discussion

To the best of our knowledge, we describe the largest group of Caucasian PCV patients to date. We found a spectrum of PCV, and propose a classification, based on a previous description of Coscas et al. [7]. In the current Caucasian patient cohort, we were able to distinguish 3 types of PCV: the first and largest group of patients had PCV within the context of typical drusen-associated nAMD, with the presence of ≥ 5 drusen with a diameter of ≥ 63 μm in the affected and fellow eye (type A PCV); the second group comprised patients with PCV in combination with a non-polypoidal type 1 CNV or BVN component, but without drusen as typical signs of AMD (type B PCV); the third group included patients with idiopathic PCV with neither drusen nor signs of non-polypoidal type 1 CNV or BVN (also not in the fellow eye; type C PCV). While drusen-associated PCV type A generally has a normal to thin choroid without pachyvessels, types B and C may also have a pachychoroid in a considerable proportion (but not all) of the cases [7, 9]. However, a remarkably high number of patients who received SD-OCT in our study had a thin choroid, also in cases of type B and C PCV.

Our study reveals additional differences between Caucasian and Asian PCV patients. PCV in our study tends to manifest at a slightly older age (73 years versus 65–73 years in Asians) [12, 13, 32–35]. A male preponderance (42–83%) is reported in Asians, while in the current cohort the opposite was found, with a female preponderance of 66% [12, 13, 32–35], which is also in contrast to some previous studies on PCV in Caucasians, in whom men and women were in general evenly represented [8, 36]. PCV accounts for about 22–55% of cases manifesting as nAMD in Asians, compared with 8.7% of Caucasian population [15], but some authors have described a prevalence of PCV in Caucasians up to 31.9%. Polypoidal lesions in Asians are often located in the macular region (78–94%) rather than in the peripapillary region (6–14%), while this difference is less pronounced in Caucasians (75% in the macula and 19% in the peripapillary region). [12, 13, 32, 33, 35] Most remarkably, half of the patients in the current Caucasian PCV study showed clinical similarities with nAMD in Caucasians, including the following: age [37, 38], laterality [33, 38], presence of drusen, and presence of a type 1 neovascularization [39, 40]. This is in line with the increasingly established idea of PCV being a variant manifestation of a type 1 neovascularization within the spectrum of AMD [4, 11, 15], which is also in line with findings from Balaratnasingam et al., who consider PCV/pachychoroid neovasculopathy to be part of the nAMD spectrum [3]. Remarkably, drusen have also been found to be present in 14.7–27% of PCV patients in previous studies [7, 15]. There is ample evidence that many, if not all, cases of PCV actually constitute an aneurysmal type 1 macular neovascularization, that is located between Bruch’s membrane and the RPE, not in the choroid. [3, 4] However, this is still subject of controversy, and a recent expert panel effort could not reach consensus on the terminology of PCV versus aneurysmal type 1 neovascularization, because of differences in opinion on whether polypoidal structures represent ‘simple’ aneurysmal dilations or more complex structures [5]. Based on our findings on multimodal imaging, we also postulate that most cases of PCV are actually aneurysmal type 1 neovascularization. Still, in several patients in our study we had evidence of a potential connection of these lesions to the underlying choroid through an interrupted Bruch’s membrane.

Traditionally, AMD by definition is associated with some kind of drusen, whether these are ‘traditional’ sub-RPE drusen and/or subretinal drusenoid deposits (also known as reticular pseudodrusen) [18, 41, 42]. However, we have previously shown that there is a spectrum of subretinal neovascularization in the macula in elderly without drusen in the affected eye as well as fellow eye [19]. In the current study on PCV, we had similar observations: 50% of PCV patients did not show drusen in the affected and fellow eye. Remarkably, 88.2% of patients with sufficiently gradable multimodal imaging had a choroidal thickness below 350 μm, without any drusen, and often without pachyvessels. This indicates that it is not uncommon for Caucasians to have PCV with a thin-to-normal choroidal thickness, and no drusen as signs of ‘typical’ AMD. Other studies on PCV in Caucasians have also described an absence of drusen in a considerable proportion of patients [7, 36]. The pathophysiology of these nAMD and PCV subtypes without drusen may therefore be different from drusen-associated AMD. For instance, pachychoroid neovasculopathy and pachychoroid-associated PCV may be pathophysiologically and clinically more close to central serous chorioretinopathy [9, 43]. In our study, only 6 out of 22 patients with OCT imaging of sufficient quality showed pachyvessels. The mean subfoveal choroidal thickness in these patients was thin (245 μm), and only 2 out of the 22 patients with SD-OCT measurements had a pachychoroid of > 350 μm (396 and 420 μm, respectively) [31]. Therefore, we postulate that there is a third form of PCV, which is associated with a relatively thin choroid, and is neither associated with drusenoid AMD, nor with pachychoroid and/or pachyvessels.

All PCV patients, regardless of whether there is a background of drusen or pachychoroid, had in common that they present with a neovascular and/or polypoidal degeneration at an age that is on average well over 70 (73 years in the current study). Hence, it seems rather arbitrary to exclude these forms of age-related neovascular degeneration of the macula without drusen (and without pachychoroid) from the definition of AMD. It is not always possible to assess whether these age-related macular neovascularizations have a background of drusenoid AMD, pachychoroid, or neither of the aforementioned, while virtually all forms require some form of anti-VEGF therapy. Thus, it may be reasonable to consider these 3 subtypes as different clinical manifestations of age-related macular neovascularization, or nAMD, when other causes such as myopic neovascularization and multifocal choroiditis have been excluded. However, we and others have hypothesised that the pathogenesis and genetic background of macular neovascularization associated with drusenoid AMD is different from that of for instance pachychoroid-associated macular neovascularization (pachychoroid neovasculopathy, with or without a polypoidal component) [3, 4, 7, 19]. The clinical subtype of nAMD and PCV could have therapeutic consequences: for instance, patients with PCV have been described to have a higher likelihood of resistance to anti-VEGF treatment in some studies [8, 9].

We have previously found differences in complement levels and genetic risk factors between central serous chorioretinopathy and AMD [44, 45]. In an Asian population, single nucleotide polymorphisms in chromosomal regions of the *complement factor H*, *age-related maculopathy susceptibility 2* (*ARMS2*), and *high-temperature requirement factor A1* genes have been associated both with PCV and nAMD [46]. However, an association between the rs10490924 variant of the *ARMS2* gene and PCV in combination with a BVN was recently found, suggesting that PCV in combination with BVN shares genetic features with nAMD, which is in contrast with PCV without BVN [47, 48]. Little is known on genetic associations in Caucasian PCV patients [15]. While there is clinical and genetic evidence of common disease determinants and pathways between PCV and AMD, we hypothesise that these disease risk factors and pathways may diverge considerably between drusen-associated and non-drusen-associated PCV. Drusen have been described to be a hallmark of complement activation, whereas other factors such as a pachychoroid background may be more important in non-drusen-associated PCV [49]. Likewise, there may be significant differences in (patho)genetic background between PCV associated with drusen, PCV with non-polypoidal type 1 CNV or BVN but without drusen, and idiopathic solitary PCV without drusen.

In the present analysis of Caucasian PCV cases, we observed a good anatomical response to treatment. Here, the most frequently performed initial therapy was conventional thermal laser (36%), closely followed by combination therapy of PDT and anti-VEGF injections (33%), and PDT monotherapy (17%). In 51% of patients, the initially performed therapy showed good anatomical recovery (Table 3, Supplementary Table 3). However, our study has several limitations, inherent to the fact that this was a retrospective study that also included follow-up of PCV patients who were initially phenotyped before the advent of novel imaging modalities such as SD-OCT. It should be noted that no firm conclusions on clinical outcome can be drawn from this analysis, given the inclusion bias (conventional laser is only used in extramacular polypoidal lesions, and nowadays performed only rarely), and the retrospective nature of this study. Due to the relatively small number of included patients, possible differences between both treatment regimens and between disease subtypes could not be studied. Moreover, choroidal thickness should be taken into account in more detail in the future, since OCT scans of sufficient quality were only available for a minority of our patients, also because of the fact that the majority of our patients have been included in the pre-(SD-)OCT era. OCT angiography could also provide additional information on the neovascular component of lesions to be studied. However, currently available devices would not be optimal to assess the PCV component, since direction of flow and low flow phenomena cannot be determined reliably [50]. Assessing pachyvessels in our study also proved to be challenging, without both the availability of a clear cut-off value for the definition of pachyvessels, and the difficulty of distinguishing several choroidal layers in patients with a relatively thin choroid [3].

Collectively, these findings support the hypothesis that PCV in Caucasians is phenotypically and presumably pathophysiologically more associated with AMD than in Asians with PCV, in whom a pachychoroid background is more common [7–9]. In Caucasian PCV, a spectrum of 3 subtypes of PCV can be distinguished (A, B, and C), out of which only type A PCV has a background of typical, drusen-associated AMD. Future prospective studies should aim at unravelling the underlying (patho)genetic background and optimal treatment in these 3 subgroups of PCV in the Caucasian population.

## Electronic supplementary material

ESM 1 (DOCX 13 kb)
