# Supplementary material for: The spectrum of polypoidal choroidal vasculopathy in Caucasians: clinical characteristics and proposal of a classification
Source: Graefes Arch Clin Exp Ophthalmol. 2020 Aug 18;259(2):351–61. doi: 10.1007/s00417-020-04844-z (PMC7843551; doi:10.1007/s00417-020-04844-z)
Supplement: Supplementary file 1 — (DOCX 13 kb) [file 417_2020_4844_MOESM1_ESM.docx]

**Supplementary Table 1: Additional therapy for all polypoidal choroidal vasculopathy patients and its effect and recurrence after 6 months**

| **Additional therapy** | **Complete resolution of subretinal fluid** | No recurrence from polypoidal lesion | Recurrence from same polypoidal lesion | Recurrence from new polypoidal lesion |
| --- | --- | --- | --- | --- |
| Anti-VEGF, n (%) | 3 (27.3) | 9 (81.8) | 2 (18.2) | - |
| PDT, n (%) | 1 (25) | 4 (100) | - | - |
| Laser, n (%) | 3 (60) | 4 (80) | 1 (20) | - |
| Anti-VEGF & PDT, n (%) | 2 (66.7) | 3 (100) | - | - |
| Anti-VEGF & laser, n (%) | 0 (0) | 1 (50) | 1 (50) | - |

Abbreviations: n, number of eyes; nAMD, PDT, photodynamic therapy; VEGF, vascular endothelial growth factor.
